# Uncontrolled metabolic syndrome associated with poorer sleep quality in adults in rural areas: a secondary analysis

**DOI:** 10.1210/jendso/bvag251

**Published:** 2026-10-28

**Authors:** Mary C Hastert, Danny Kurz, Catherine Siengsukon, Debra K Sullivan, Christie Befort

**Affiliations:** Department of Internal Medicine, The University of Kansas Medical Center, Kansas City, KS 66160, USA; Department of Population Health, The University of Kansas Medical Center, Kansas City, KS 66160, USA; Department of Physical Therapy, Rehabilitation Science, and Athletic Training, The University of Kansas Medical Center, Kansas City, KS 66160, USA; Department of Dietetics and Nutrition, The University of Kansas Medical Center, Kansas City, KS 66160, USA; Department of Population Health, The University of Kansas Medical Center, Kansas City, KS 66160, USA

**Keywords:** sleep quality, metabolic syndrome, rural

## Abstract

**Objective:**

To examine whether clinical management of metabolic syndrome (MetS) is associated with sleep health among adults receiving care in rural primary care settings.

**Methods:**

This cross-sectional analysis used baseline data from adults (age 20-75 years; body mass index (BMI) 30-45 kg/m^2^) enrolled in the RE-POWER trial, a pragmatic behavioral weight loss intervention implemented in rural primary care clinics. Sleep quality was assessed using the Pittsburgh Sleep Quality Index (PSQI). MetS was defined according to American Heart Association/National Heart, Lung, and Blood Institute (AHA/NHLBI) criteria and categorized as: (1) no-MetS, (2) controlled MetS (criteria met via medication use), and (3) uncontrolled MetS (criteria met via abnormal laboratory values). Linear mixed models evaluated whether MetS status predicted global PSQI, PSQI subcomponents, and sleep duration, adjusting for age, sex, and BMI.

**Results:**

Among 1406 adults (mean age 54.6 ± 11.8 years; 76.8% female), those with uncontrolled MetS (n = 634) had significantly worse global PSQI, sleep latency, and sleep disturbance scores compared with adults without MetS (n = 483) (*P* < .05). Sleep outcomes did not differ between adults with controlled MetS (n = 289) and those without MetS (*P* > .05).

**Conclusion:**

Poorly controlled MetS was associated with poorer sleep quality, whereas clinically controlled MetS was not. Future research is needed to determine if effective clinical management of MetS may confer benefits for sleep health in rural populations.

An estimated one-third of adults in the United States have metabolic syndrome (MetS) (1), a clustering of cardiometabolic risk factors that includes central obesity, impaired glucose metabolism, elevated blood pressure, and dyslipidemia (2). While each risk factor carries independent risk of developing cardiometabolic diseases, their presence together is synergistic and poses greater risk of developing type 2 diabetes and cardiovascular diseases compared to when found alone (3). Additionally, MetS is associated with poorer self-reported sleep health (4) and clinically diagnosed sleep disorders such as insomnia (5) and obstructive sleep apnea (6). The relationship between MetS and poor sleep may be driven by shared underlying biological mechanisms such as glucose dysregulation and insulin resistance (7-9), as well as an increased risk of anxiety and stress associated with MetS (10, 11) which may hinder the ability to achieve a restful sleep.

Uncontrolled MetS and sleep disorders both present significant financial burdens for individual patients and the US healthcare system. Adults with MetS have up to 120% greater healthcare expenditure compared to those without MetS, and it is estimated that each individual MetS risk factor raises costs by ∼24% (12). Similarly, the presence of a sleep disorder costs individuals an estimated additional ∼$11 000 per year, and sleep disorders are responsible for an estimated ∼$90 billion cost burden on the US healthcare system (13). Addressing both conditions concurrently may offer a cost-effective strategy to improve health outcomes and reduce healthcare costs.

Existing research on concurrent improvements in MetS and sleep health has largely focused on interventions that target underlying sleep disorders and then assess downstream metabolic outcomes. For example, adherence to continuous positive airway pressure therapy for obstructive sleep apnea has been shown to improve glycemic control and blood pressure (14, 15). Other nonpharmacological sleep interventions, such as exercise and sleep counseling, have demonstrated improvements in sleep quality but have been less effective in producing clinically significant improvements in metabolic outcomes (16). In contrast, it remains unclear whether effective management of MetS can directly improve sleep outcomes and whether MetS treatment could serve as an optimal initial strategy for concurrently improving both metabolic and sleep health. Clinical management of MetS, as outlined in the American Heart Association/National Heart, Lung, and Blood Institute (AHA/NHLBI) guidelines, involves a combination of lifestyle modification and/or pharmacological treatment to address individual MetS risk factors (17). Adherence to these guidelines has been shown to effectively treat MetS and sustain remission (18), yet the extent to which such management influences sleep outcomes remains unknown.

The potential for clinical management of MetS to concurrently improve sleep health is particularly relevant in rural populations where adults experience significant disparities in both metabolic and sleep health, as well as healthcare access. Data from 669 978 adults participating in the Behavioral Risk Factor Surveillance System indicate a higher prevalence of short sleep duration (<7 hours per night) among rural compared with urban residents (19). In addition, adults living in rural areas report greater difficulty initiating and maintaining sleep (20). Rural residence is also associated with a 23% increased risk of developing MetS (21), alongside higher rates of obesity (22), type 2 diabetes, and cardiovascular disease (23). These disparities are compounded by structural limitations in healthcare access, as rural adults rely more heavily on primary care services (24), experience higher rates of uninsurance (25), and greater use of Medicare and Medicaid (26, 27). Consequently, effective strategies for managing both MetS and sleep health are especially needed in rural primary care settings. Therefore, this cross-sectional analysis aimed to examine whether the presence of MetS and adherence to guideline-based clinical management are associated with self-reported sleep outcomes among rural adults receiving primary care.

## Methodology

### Overview

This is a baseline analysis from a 24-month weight loss intervention, the Rural Engagement in Primary Care for Optimizing Weight Reduction (RE-POWER) trial, funded through the National Institutes of Health. The full protocol, recruitment, and outcome details have been previously published (28-30). Briefly, the RE-POWER trial was a 3-armed, multi-component lifestyle intervention developed to represent realistic weight management practices in rural healthcare clinics. The RE-POWER trial was approved by institutional review boards at the University of Kansas Medical Center and the VA Nebraska-Western Iowa Health Care System, and all participants provided written informed consent.

### Eligibility criteria and recruitment

Participants were adults aged 20 to 75 years of age with a body mass index (BMI) between 30 and 45 kg/m^2^ who resided in a rural area according to the Rural-Urban Commuting Area Codes (31). Participants were recruited from primary care clinics in Kansas, Nebraska, Wisconsin, and Iowa. Exclusion criteria were as follows: (i) history of bariatric surgery or planned bariatric surgery within 2 years; (ii) myocardial infarction, stroke, or new cancer diagnosis in the last 6 months; (iii) pregnancy in the last 6 months or planned within the next 2 years; (iv) currently lactating; (v) weight loss was contraindicated as determined by their primary care physician; and (vi) plans to leave their current rural primary care clinic within 2 years of the trial.

### Assessed outcomes

Sociodemographic characteristics and comorbid medical conditions were self-reported and physical activity was measured with the Modifiable Activity Questionnaire (MAQ) scored as Metabolic Equivalent of Task (MET) hours per week by multiplying time spent in physical activities by the estimated MET and summing across all activities (32). Lists of current medications were collected via phone interview with a member of the research team. Sleep quality was assessed using the Pittsburg Sleep Quality Index (PSQI) (33) completed independently. PSQI outcomes included the global PSQI score, with a possible score from 0 to 21 with a score > 5 indicating poor sleep health; the 7 subcomponents, scored 0 to 3 (sleep quality, latency, duration, efficiency, disturbance, medication, and dysfunction); and the reported average sleep duration in hours. To explore potential confounding by obstructive sleep apnea (OSA), 2 items on the PSQI disturbance subscale were used to measure OSA-related symptoms in the absence of data on a diagnosis of OSA. “Frequent snorer” and “frequent breathing difficulty” were defined as “cough or snore loudly” ≥ 3 times per week and “cannot breathe comfortably” ≥ 3 times per week, respectively. These items have been associated with sleep disordered breathing measured by device-based respiratory disturbance index event rates (34). Presence of MetS was assessed via laboratory and clinic-based values, including blood pressure (mmHg), triglycerides (mg/dL), high-density lipoprotein cholesterol (HDL-C; mg/dL), and fasting glucose (mg/dL).

### Classification of groups

MetS presence was defined according to the criterion in the scientific statement from the AHA/NHLBI (17). Presence was determined by meeting any 3 out of 5 criterion: elevated waist circumference (≥40 inches in men or ≥35 inches in women OR BMI classified as obesity, ≥30 kg/m^2^), elevated triglycerides (≥150 mg/dL OR on drug treatment for elevated triglycerides), reduced HDL-C (<40 mg/dL in men and <50 mg/dL in women OR on drug treatment for reduced HDL-C), elevated blood pressure (>130 mm Hg systolic blood pressure or >85 mm Hg diastolic blood pressure OR antihypertensive drug treatment in a patient with history of hypertension), and elevated fasting glucose (>100 mg/dL OR on drug treatment for elevated glucose) (17). Participants were classified into 3 groups for analysis. Group 1 was without MetS. Group 2 was present with MetS according to AHA/NHLBI guidelines (17) if they were taking the relevant medications (“controlled MetS” group). Group 3 was present with MetS according to AHA/NHLBI guidelines (17) from qualifying lab values (“uncontrolled MetS” group).

### Statistical analysis

All analyses were completed using SPSS Statistics 27 (2020 Armonk, NY: IBM Corp). Mean and SD and descriptive statistics were used to describe sociodemographic and medical characteristics. ANOVA and chi square analyses evaluated between-group differences in sociodemographic characteristics, medical characteristics, OSA-related symptoms, and physical activity as potential confounders. Nine separate linear mixed analyses were used to evaluate whether MetS status predicted sleep outcomes; MetS status was the predictor variable and global PSQI, subcomponents of the PSQI, and mean sleep duration were the dependent variables. Due to their known influence/association with MetS and sleep outcomes, and based on observed significant differences across MetS groups, age, sex, and BMI were included as covariates (19, 35-37). Although OSA-related symptoms did not significantly differ across MetS groups, due to the known connection between OSA and metabolic syndrome, sensitivity analyses were conducted by removing participants (n = 295) who reported the specific OSA-related symptom of frequent snoring. Results were reported as mean plus 95% CI with a significance level set at .05.

## Results

The baseline sample size of the RE-POWER trial was 1407. Included in this analysis were 1406 participants with blood values available at baseline.

### Sociodemographic and medical characteristics

Participant sociodemographic variables are presented in Table 1. The total sample was 54 ± 11 years of age and primarily consisted of female patients (76%). The no-MetS group was the youngest with the highest percentage of females and those employed full-time, whereas the controlled MetS group was the oldest with the highest percentage of married individuals. The uncontrolled MetS group had the highest body weight and highest percentage of males.

**Table 1:** Sociodemographic characteristics of the total sample and by group

Variable, mean ± SD	No-MetS, Group 1 (n = 483)	Controlled MetS, Group 2 (n = 289)	Uncontrolled MetS, Group 3 (n = 634)	Total (n = 1406)	*P* value
Age, years	50.2 ± 12.0	59.2 ± 9.7	55.9 ± 11.5	54.6 ± 11.8	<.001
Weight, kilograms	99.5 ± 13.9	102.6 ± 15.6	105.6 ± 16.1	102.9 ± 15.5	<.001
BMI, kg/m^2^	36.1 ± 3.8	36.7 ± 4.2	37.3 ± 3.9	36.8 ± 4.0	<.001
Sex, female*^a^*	420 (87.0)	211 (73.0)	449 (70.8)	1080 (76.8)	<.001
Rurality*^a^*					.06
*Isolated rural*	244 (50.5)	129 (44.6)	285 (45.0)	658 (46.8)	
*Small rural*	75 (15.5)	45 (15.6)	130 (20.5)	250 (17.8)	
*Large rural*	164 (34.0)	115 (39.8)	219 (34.5)	498 (35.4)	
Race/Ethnicity, White, non-Hispanic*^a^*	465 (96.3)	280 (96.9)	607 (95.7)	1352 (96.2)	.90
Marital status*^a^*					.07
*Married*	389 (80.5)	231 (79.9)	477 (75.2)	1097 (78.0)	
*Single*	94 (19.5)	58 (20.1)	157 (24.8)	309 (22.0)	
Education*^a^*					.75
*High school graduate or less*	92 (19.0)	60 (20.8)	144 (22.7)	296 (21.1)	
*Some college*	242 (50.1)	145 (50.2)	304 (47.9)	691 (49.1)	
*Bachelor's degree*	97 (20.1)	49 (17.0)	116 (18.3)	262 (18.6)	
*Graduate degree*	52 (10.8)	35 (12.1)	70 (11.0)	157 (11.2)	
Employment status*^a^*					<.001
*Employed full-time*	323 (66.9)	139 (48.1)	318 (50.2)	780 (55.5)	
*Employed part-time*	73 (15.1)	36 (12.5)	101 (15.9)	210 (14.9)	
*Retired*	55 (11.4)	88 (30.4)	156 (24.6)	299 (21.3)	
*Other*	32 (6.6)	29 (9.0)	59 (9.3)	117 (8.3)	
Physical activity (MET-hrs/wk)	7.8 ± 12.2	7.2 ± 10.4	7.0 ± 16.7	7.3 ± 14.1	.65

Abbreviations: BMI, body mass index; MET, Metabolic Equivalent of Task; MetS, metabolic syndrome.

^*a*^Reported as n (% sample).

Participant medical conditions and medications are reported in Table 2. As expected, the groups significantly differed in the presence of hypertension, hypercholesterolemia, and diabetes (*P* < .001), as well as cardiovascular disease (*P* < .001). They did not differ with regard to depression (*P* = .56). All groups significantly differed in use of medications (*P* < .001). Roughly half of the total sample self-reported taking blood pressure–lowering medications, and a third self-reported taking cholesterol-lowering medications. The groups significantly differed in all abnormal lab values, and the most common abnormal value was low HDL-C (∼45% of the total sample).

**Table 2:** Medical conditions, medications, and abnormal lab values related to metabolic syndrome

Variable, n (% sample)	No-MetS, Group 1 (n = 483)	Controlled MetS, Group 2 (n = 289)	Uncontrolled MetS, Group 3 (n = 634)	Total (n = 1406)	*P* value
Medical conditions					
*Hypertension*	110 (22.8)	181 (62.6)	351 (55.4)	642 (45.7)	<.001
*Hypercholesterolemia*	29 (6.0)	217 (75.1)	320 (50.5)	566 (40.3)	<.001
*Depression*	185 (38.3)	121 (41.9)	244 (38.5)	550 (39.1)	.56
*Diabetes*	21 (4.3)	82 (28.4)	233 (36.8)	336 (23.9)	<.001
*Cancer history*	36 (7.5)	29 (10.0)	82 (12.9)	147 (10.5)	.012
*Cardiovascular disease*	12 (2.5)	20 (6.9)	59 (9.3)	91 (6.5)	<.001
OSA-related symptoms					
*Frequent snorer ≥ 3×/week*	87 (18.0)	60 (20.8)	148 (23.3)	295 (21.0)	.10
*Frequent difficulty breathing ≥ 3×/week*	16 (3.3)	15 (5.2)	30 (4.7)	61 (4.3)	.37
Medications					
*Cholesterol-lowering*	0 (0.0)	221 (76.5)	289 (45.6)	510 (36.3)	<.001
*Glucose-lowering*	7 (1.4)	63 (21.8)	194 (30.6)	264 (18.8)	.006
*Blood pressure–lowering*	125 (25.9)	209 (72.3)	399 (62.9)	733 (52.1)	.005
Abnormal lab values					
*Triglycerides*	26 (5.4)	38 (13.1)	430 (67.8)	494 (35.1)	<.001
*HDL-C*	101 (20.9)	67 (23.3)	471 (74.3)	639 (45.4)	<.001
*Blood pressure*	12 (2.5)	45 (15.6)	328 (51.7)	385 (27.4)	<.001
*Glucose*	134 (27.7)	54 (18.7)	378 (59.6)	566 (40.3)	<.001

Abbreviations: HDL-C, high-density lipoprotein cholesterol; MetS, metabolic syndrome; OSA, obstructive sleep apnea.

### Differences in sleep outcomes by group

Comparison of the global PSQI, PSQI subscores, and sleep duration between groups are presented in Table 3. A global PSQI score >5 indicates poor sleep health (33) and all groups presented with a mean global PSQI score greater than 7. There was a significant difference on the global PSQI score between the no-MetS group vs the uncontrolled MetS group (*P* < .05). Within the 7 subscores of the PSQI, there was a significant difference in sleep latency (*P* < .05) and sleep disturbance (*P* < .05) between the no-MetS vs uncontrolled MetS group. There was no significant difference on the global PSQI score between no-MetS vs controlled MetS or between uncontrolled MetS vs controlled MetS (*P* > .05). Across all groups, there were no significant differences in self-reported sleep duration (*P* > .05).

**Table 3:** Results of the PSQI among groups

	Between-group net difference					
	No-MetS (Group 1)	Controlled MetS (Group 2)	Uncontrolled MetS (Group 3)	Group 1 vs 2	Group 1 vs 3	Group 2 vs 3
Global PSQI, (0-21)	7.36 (7.02, 7.71)*^a^*	7.75 (7.31, 8.18)	7.86 (7.57, 8.15)*^a^*	−0.38 (−0.94, 0.18)	−0.49 (−0.95, −0.04)*^a^*	−0.11 (−0.63, 0.40)
PSQI subcomponents, (0-3)						
*Sleep quality*	1.11 (1.04, 1.17)	1.19 (1.11, 1.27)	1.19 (1.13, 1.24)	−0.08 (−0.18, 0.03)	−0.08 (−0.16, 0.00)	0.00 (−0.10, 0.09)
*Sleep latency*	1.09 (1.00, 1.18)*^a^*	1.17 (1.06, 1.28)	1.22 (1.14, 1.30)*^a^*	−0.08 (−0.23, 0.07)	−0.13 (−0.25, −0.01)*^a^*	−0.05 (−0.19, 0.08)
*Sleep duration*	1.30 (1.21, 1.39)	1.29 (1.18, 1.41)	1.27 (1.19, 1.35)	0.01 (−0.15, 0.16)	0.03 (−0.10, 0.15)	0.02 (−0.12, 0.16)
*Sleep efficiency*	0.75 (0.66, 0.84)	0.72 (0.61, 0.83)	0.78 (0.71, 0.86)	0.03 (−0.11, 0.18)	−0.03 (−0.15, 0.09)	−0.06 (−0.20, 0.07)
*Sleep disturbance*	1.43 (1.38, 1.49)*^a^*	1.47 (1.40, 1.54)	1.52 (1.48, 1.57)*^a^*	−0.04 (−0.13, 0.05)	−0.09 (−0.16, −0.02)*^a^*	−0.05 (−0.13, 0.03)
*Sleep medication*	0.66 (0.55, 0.76)	0.82 (0.69, 0.96)	0.77 (0.68, 0.86)	−0.17 (−0.35, 0.01)	−0.11 (−0.26, 0.03)	0.05 (−0.11, 0.22)
*Sleep dysfunction*	1.03 (0.96, 1.09)	1.08 (1.00, 1.16)	1.10 (1.05, 1.16)	−0.05 (−0.16, 0.05)	−0.08 (−0.16, 0.01)	−0.02 (−0.12, 0.07)
Sleep duration, hours	6.65 (6.54, 6.76)	6.67 (6.53, 6.81)	6.70 (6.60, 6.79)	−0.02 (−0.20, 0.16)	−0.05 (−0.20, 0.10)	−0.03 (−0.19, 0.14)

Values are mean (95% CI). Adjusted for sex, age, and BMI.

Abbreviations: BMI, body mass index; MetS, metabolic syndrome; PSQI, Pittsburgh Sleep Quality Index.

^*a*^Indicates a significant net difference (*P* < .05) between groups.

Sensitivity analyses removing participants who reported frequent snoring revealed similar patterns of significance. There remained a significant difference on the global PSQI score between the no-MetS vs uncontrolled MetS groups (*P* < .05). There was also a significant difference in sleep latency (*P* < .05) and a trend toward significance for sleep disturbance (*P* = .08) between the no-MetS vs uncontrolled MetS groups. There were no significant differences between no-MetS and controlled MetS.

## Discussion

This is the first study to evaluate whether proper clinical management of MetS influences sleep health in rural primary care. In this sample of 1406 adults aged 20 to 75 years living in rural communities, adults with uncontrolled MetS had significantly worse sleep quality, sleep latency, and sleep disturbances in comparison to those without MetS. In contrast, participants with controlled MetS showed no significant difference in sleep outcomes compared to those without MetS. Regardless of MetS status, all groups reported poor sleep quality and duration.

Our findings indicate that poor clinical management of MetS is associated with modestly worse sleep quality compared with no-MetS, while those with controlled MetS have similar sleep outcomes as those with no-MetS. These results point to the need for additional studies to confirm whether or not the clinical management of underlying MetS may offer a concurrent benefit for sleep health. However, these associations should be interpreted cautiously, as the present study is observational in nature and cannot establish causality.

Emerging interventional evidence provides preliminary support for our study findings. For example, a 2023 randomized, placebo-controlled trial by Bruno et al demonstrated that 12 months of metformin therapy in adults with MetS significantly improved total sleep time and sleep efficiency (38). Similarly, a 6-month pilot trial reported improvements in self-reported sleep duration following a lifestyle intervention among adults with MetS (39). While these early findings are promising, the current literature remains limited in scope and methodological rigor. Rigorous intervention trials integrating lifestyle and pharmacological management of MetS and incorporating objective sleep measures are needed to determine whether treating MetS causally improves sleep health in adults with and without diagnosed sleep disorders.

There are also likely biological, nutritional, and sociodemographic mechanistic pathways for the association between MetS management and sleep quality that our study was unable to capture. Biological factors associated with MetS, such as insulin resistance and adipose dysfunction, promote chronic inflammation, which, along with heightened sympathetic activity, impairs sleep quality (40). Conversely, poor sleep converges on cardiometabolic pathways as it worsens insulin sensitivity and glucose regulation, disrupts appetite hormones, raises sympathetic activity and blood pressure, and promotes endothelial dysfunction via inflammatory signaling. OSA and other sleep disorders may reinforce this cycle (41). Poor diet quality may also be an important mechanism contributing to worse sleep quality. Highly processed, calorie-dense food lacking in micronutrients leads to inadequate tryptophan and melatonin synthesis and circadian misalignment, resulting in higher sleep latency and worse overall sleep quality (42). Sleep restriction, in turn, downregulates satiety hormones and upregulates hunger signals and thereby promotes higher consumption of highly processed, calorie-dense foods. Additionally, sociodemographic factors such as age, socioeconomic status, and marital status may play a mediating role through, for example, availability of financial resources and levels of stress. Identification of factors that mediate the association between MetS management and sleep may guide more specific, targeted clinical interventions and help clinicians provide early screening and treatment for patients at high risk for concurrent MetS and sleep disorders.

Regardless of MetS status or clinical management, rural adults in our sample reported poor sleep health. Mean global PSQI scores exceeded 7 across all groups, indicating poor sleep quality (33), and average sleep duration was approximately 6 hours per night—below the National Sleep Foundation's recommended 7 to 9 hours (43). These findings are consistent with prior observational studies showing that 20% to 30% of rural US adults report short sleep duration (44-46). Addressing poor sleep in rural populations is a public health priority, given its adverse effects on metabolic health (47, 48) and its economic consequences, including reduced occupational productivity and increased healthcare costs (49, 50). These impacts may be especially pronounced in rural communities, where physically demanding agricultural and manufacturing occupations are common and rates of private health insurance are lower (25-27). Interventions targeting rural-urban sleep disparities may therefore yield both health and economic benefits.

This study has several strengths and limitations. A major strength is the large sample size as the inclusion of 1406 participants provided sufficient power to detect group differences between controlled and uncontrolled MetS status. Participants were drawn from 4 Midwestern states and represented a range of rurality, which enhances the generalizability of findings within rural populations. Several limitations should also be acknowledged. The cross-sectional design and reliance on self-reported measures limit causal inference, and reverse causality is possible with poor quality sleep contributing to poor management of MetS. In addition, we were not able to control for some important potential confounders, such as a diagnosis of OSA, other sleep disorders, severity of mental health, or other residual confounders. In addition, medical conditions and medication use were self-reported, and minor discrepancies were observed. For example, 6% more participants reported taking antihypertensive medication than reported a diagnosis of hypertension. Although such discrepancies may be clinically marginal, MetS classification based on the AHA/NHLBI criteria relies on medication use for 4 of the 5 components. As a result, accurate classification of MetS is dependent on the validity of self-reported medication use, which was collected via guided interview. This study was unable to examine potential mediating mechanisms underlying the association between appropriate MetS management and more favorable sleep outcomes. Finally, generalizability of the findings is limited to predominantly White, rural Midwestern adults with obesity who were enrolled in a primary care–based weight loss trial. Future research should extend the cross-sectional literature by employing prospective designs to evaluate how clinical management of MetS influences objective sleep outcomes, with particular attention to biological mechanisms and inclusion of adults with comorbid sleep disorders.

## Conclusion

Our findings indicate that, among adults living in rural communities, those with uncontrolled MetS experienced poorer sleep quality compared with adults without MetS, whereas sleep quality among adults with controlled MetS were comparable to those without MetS. These results point to the need for prospective and interventions studies to evaluate whether clinical management of underlying MetS may offer a concurrent benefit for sleep health.

Routine assessment of sleep health in rural primary care settings may be beneficial, both to identify sleep-related disparities and to better understand their influence on cardiometabolic outcomes.

## Data Availability

Some or all datasets generated during and/or analyzed during the current study are not publicly available but are available from the corresponding author on reasonable request.

## Abbreviations

AHA: American Heart Association
BMI: body mass index
HDL-C: high-density lipoprotein cholesterol
MetS: metabolic syndrome
NHLBI: National Heart, Lung, and Blood Institute
OSA: obstructive sleep apnea
PSQI: Pittsburgh Sleep Quality Index
RE-POWER: Rural Engagement in Primary Care for Optimizing Weight Reduction
